# TheraSphere Yttrium-90 Glass Microspheres Combined With Chemotherapy Versus Chemotherapy Alone in Second-Line Treatment of Patients With Metastatic Colorectal Carcinoma of the Liver: Protocol for the EPOCH Phase 3 Randomized Clinical Trial

**DOI:** 10.2196/11545

**Published:** 2019-01-17

**Authors:** Nikhil Chauhan, Mary F Mulcahy, Riad Salem, Al B Benson III, Eveline Boucher, Janet Bukovcan, David Cosgrove, Chantal Laframboise, Robert J Lewandowski, Fayaz Master, Bassel El-Rayes, Jonathan R Strosberg, Daniel Y Sze, Ricky A Sharma

**Affiliations:** 1 Research and Development BTG International group companies London United Kingdom; 2 Division of Hematology and Oncology Department of Medicine Robert H Lurie Comprehensive Cancer Center of Northwestern University Chicago, IL United States; 3 Section of Interventional Radiology Department of Radiology Robert H Lurie Comprehensive Cancer Center of Northwestern University Chicago, IL United States; 4 Division of Transplant Surgery Department of Surgery Robert H Lurie Comprehensive Cancer Center of Northwestern University Chicago, IL United States; 5 Northwestern Medical Group Feinberg School of Medicine Northwestern Memorial Hospital Chicago, IL United States; 6 Division of Medical Oncology Vancouver Cancer Center Compass Oncology Vancouver, WA United States; 7 Winship Cancer Institute Emory University Atlanta, GA United States; 8 Department of Hematology and Medical Oncology Emory University School of Medicine Atlanta, GA United States; 9 H Lee Moffitt Cancer Center Tampa, FL United States; 10 Interventional Radiology Stanford University Medical Center Stanford, CA United States; 11 National Institute for Health Research University College London Hospitals Biomedical Research Centre UCL Cancer Institute London United Kingdom

**Keywords:** colorectal neoplasms, neoplasm metastasis, microspheres, yttrium radioisotopes, research design, clinical trial, phase III, randomized controlled trial, metastatic colorectal cancer, mCRC

## Abstract

**Background:**

Colorectal cancer is one of the most common cancers and causes of cancer-related death. Up to approximately 70% of patients with metastatic colorectal cancer (mCRC) have metastases to the liver at initial diagnosis. Second-line systemic treatment in mCRC can prolong survival after development of disease progression during or after first-line treatment and in those who are intolerant to first-line treatment.

**Objective:**

The objective of this study is to evaluate the efficacy and safety of transarterial radioembolization (TARE) with TheraSphere yttrium-90 (^90^Y) glass microspheres combined with second-line therapy in patients with mCRC of the liver who had disease progression during or after first-line chemotherapy.

**Methods:**

EPOCH is an open-label, prospective, multicenter, randomized, phase 3 trial being conducted at up to 100 sites in the United States, Canada, Europe, and Asia. Eligible patients have mCRC of the liver and disease progression after first-line chemotherapy with either an oxaliplatin-based or irinotecan-based regimen and are eligible for second-line chemotherapy with the alternate regimen. Patients were randomized 1:1 to the TARE group (chemotherapy with TARE in place of the second chemotherapy infusion and subsequent resumption of chemotherapy) or the control group (chemotherapy alone). The addition of targeted agents is permitted. The primary end points are progression-free survival and hepatic progression-free survival. The study objective will be considered achieved if at least one primary end point is statistically significant. Secondary end points are overall survival, time to symptomatic progression defined as Eastern Cooperative Oncology Group Performance Status score of 2 or higher, objective response rate, disease control rate, quality-of-life assessment by the Functional Assessment of Cancer Therapy-Colorectal Cancer questionnaire, and adverse events. The study is an adaptive trial, comprising a group sequential design with 2 interim analyses with a planned maximum of 420 patients. The study is designed to detect a 2.5-month increase in median progression-free survival, from 6 months in the control group to 8.5 months in the TARE group (hazard ratio [HR] 0.71), and a 3.5-month increase in median hepatic progression-free survival time, from 6.5 months in the control group to 10 months in the TARE group (HR 0.65). On the basis of simulations, the power to detect the target difference in either progression-free survival or hepatic progression-free survival is >90%, and the power to detect the target difference in each end point alone is >80%.

**Results:**

Patient enrollment ended in October 2018. The first interim analysis in June 2018 resulted in continuation of the study without any changes.

**Conclusions:**

The EPOCH study may contribute toward the establishment of the role of combination therapy with TARE and oxaliplatin- or irinotecan-based chemotherapy in the second-line treatment of mCRC of the liver.

**Trial Registration:**

ClinicalTrials.gov NCT01483027; https://clinicaltrials.gov/ct2/show/NCT01483027 (Archived by WebCite at http://www.webcitation.org/734A6PAYW)

**International Registered Report Identifier (IRRID):**

RR1-10.2196/11545

## Introduction

Colorectal cancer is the third most common newly diagnosed cancer and the fourth most common cause of cancer death globally [1]. Metastatic disease is observed at first diagnosis in an estimated 25% of new patients (synchronous distant metastasis) [2] and eventually develops in a further estimated 60% of patients (metachronous metastasis) [2-4]. The prognosis for metastatic colorectal cancer (mCRC) remains poor, with a 5-year survival rate around 14% [5]; the 5-year relative survival is worse for patients with metachronous metastasis than for those with synchronous metastasis (17.6% vs 7.2%) [6]. The liver is the most common site of metastasis because the blood that drains from the bowel and colon goes through the portal vein, and the circulating tumor cells are deposited in the liver [7,8]. Up to approximately 70% of metastatic patients present with mCRC to the liver at the initial diagnosis [7].

Although the outcome for patients with mCRC has improved with the rapid progress in diagnostic techniques and treatments, for most patients with mCRC, treatment is palliative rather than curative because the majority of patients are not candidates for surgical resection [9]. In mCRC patients for whom cure is not possible, potential goals of treatment are to prolong progression-free intervals, prolong life, improve quality of life, palliate symptoms, shrink tumor size, and protect the normal liver parenchyma. Patients with mCRC to the liver can achieve a median overall survival of approximately 30 months [10,11].

Most patients with mCRC receive systemic chemotherapy with or without targeted biological agents. First- and second-line systemic therapies typically include a fluoropyrimidine combined with either irinotecan (FOLFIRI regimen) or oxaliplatin (FOLFOX regimen). Biologically targeted agents include vascular endothelial growth factor inhibitors and epidermal growth factor receptor inhibitors [12]. Liver-directed therapies, that is, transarterial chemoembolization (TACE) and transarterial radioembolization (TARE), are well established in the armamentarium for treatment of metastatic disease, mostly in the salvage setting. The delivery of chemotherapy or radioactive-labeled yttrium-90 (^90^Y) microspheres via the hepatic arteries is selective to liver tumors because liver tumors are mostly perfused via the hepatic arteries, whereas normal hepatic parenchyma receives blood from the portal venous system [13].

Most mCRC patients who receive standard first-line treatment with combination regimens and treatment with targeted biological agents eventually develop either intolerance, recurrence, or progression and require second-line treatment. TARE is a treatment option that could be considered for patients with unresectable colorectal cancer and liver-dominant metastases who are refractory to chemotherapy. In such patients, prospective and retrospective studies have demonstrated that TARE is feasible and may compare favorably with standard-of-care treatment [14-16]. A rationale for the combination of TARE and systemic therapy is that liver-directed treatment with TARE will better control liver disease and systemic therapy will control extrahepatic progression and micrometastatic disease.

Here, we report the design of the EPOCH study: A Phase 3 Randomized Clinical Trial Evaluating TheraSphere in Patients with Metastatic Colorectal Carcinoma of the Liver Who Have Failed First-line Chemotherapy (trial registration: clinicaltrials.gov NCT01483027). The EPOCH study is being conducted to evaluate progression-free survival and hepatic progression-free survival in patients with mCRC when TheraSphere is added to second-line standard-of-care chemotherapy. In the second-line mCRC treatment setting, the EPOCH study is expected to be the largest study of the comparison of locoregional therapy with TARE in combination with standard-of-care systemic therapy versus standard-of-care systemic therapy alone.

## Methods

### Overview of Design

The study is being conducted in accordance with the Declaration of Helsinki. Participating institutions obtained institutional review board approval of the protocol and informed consent form and are responsible for obtaining written informed consent from patients at screening (Multimedia Appendix 1).

EPOCH is an ongoing, open-label, prospective, multicenter, randomized, phase 3 clinical trial. The objective is to evaluate the efficacy and safety of TARE with TheraSphere in patients with mCRC of the liver who have disease progression on first-line chemotherapy with either an oxaliplatin-based regimen or an irinotecan-based regimen and who are eligible for second-line chemotherapy with the alternate regimen. All patients receive chemotherapy; however, in the TARE group, TheraSphere is administered in place of the second cycle of chemotherapy, with chemotherapy subsequently resuming. A maximum of 420 patients are planned to be randomized at up to 100 sites in the United States, Canada, Europe, and Asia. The 2 primary end points are progression-free survival and hepatic progression-free survival. The study objective will be considered achieved if at least one primary end point is statistically significant. All patients are to be followed prospectively from randomization to death until the predefined number of progression-free survival events, to allow the final analysis to be conducted, have occurred.

The study commenced enrollment with a single primary end point of progression-free survival, with hepatic progression-free survival as a secondary end point. Progression-free survival is a valid surrogate end point for overall survival for mCRC patients receiving first- and second-line systemic chemotherapy with or without the inclusion of systemic targeted therapies [17,18]. The expected benefit for patients receiving a liver-directed therapy is an increased duration of liver disease control. Accordingly, the efficacy of a liver-directed treatment can be evaluated and measured by hepatic progression-free survival. A survival benefit of liver-directed treatment could occur via improvement of hepatic progression-free survival. Other liver-directed therapies, such as intra-arterial chemotherapy infusion, radiofrequency ablation, or TACE with drug-eluting beads, have demonstrated improvement of overall survival through improved control of hepatic disease [19,20]; thus, the efficacy of a liver-directed treatment may be evaluated and measured by hepatic progression-free survival [21]. To demonstrate the clinical benefit of TARE in mCRC patients with liver metastases, hepatic progression-free survival was subsequently included as a second primary end point. Progression-free survival and hepatic progression-free survival will evaluate the 2 major determinants of progression (extrahepatic factors and intrahepatic factors).

Trained clinical research associates performed a site initiation visit with investigators and their teams before the start of patient screening. Patient medical records are reviewed in a timely manner to confirm eligibility and compliance with the study protocol. Data entries on the electronic case report forms are reviewed by the clinical research associate and compared with the medical records and study protocol on an ongoing basis. An independent data monitoring committee (IDMC) was established to oversee the conduct of the study. The IDMC met periodically to review enrollment, protocol deviations, and safety events. In addition, the IDMC conducted and reviewed an initial feasibility safety analysis and will evaluate the progression-free survival data at interim analyses for consideration of stopping the study early for efficacy. The IDMC was tasked to make formal recommendations to the study sponsor based on decision rules in the IDMC charter.

### Screening and Eligibility

Screening and baseline evaluations occur from day −14 to day 0, where day 0 is the day of randomization. Demographics, medical history, medications, prior treatment history, and Eastern Cooperative Oncology Group (ECOG) Performance Status score were documented. Patients undergo physical examination and have baseline clinical laboratory tests including blood chemistry, hematology, coagulation tests, and colorectal cancer tumor marker (serum carcinoembryonic antigen). Kirsten retrovirus-associated DNA sequence (KRAS) oncogene status is determined if it is not already known. Serum pregnancy tests were conducted for women of childbearing potential. Patients must have baseline images for disease evaluation (spiral computerized tomography [CT] or magnetic resonance imaging [MRI] of the abdomen, pelvis, and chest) taken within 28 days before day 0 (randomization) when first-line chemotherapy is completed or after, and images must show measurable target tumors in the liver according to Response Evaluation Criteria In Solid Tumors (RECIST) version 1.1 [22]. Tumor burden was estimated from CT scans (visual or volumetric assessment). Inclusion and exclusion criteria are shown in Textbox 1.

Eligible patients must discontinue first-line chemotherapy and biologic agents during screening for a washout period of at least 14 days. The intended second-line chemotherapy regimen, including any biological agents, and dosages are decided. Patients complete the Functional Assessment of Cancer Therapy-Colorectal Cancer (FACT-C) quality-of-life questionnaire during the screening period.

**Inclusion and exclusion criteria for the EPOCH study.**

**Inclusion criteria**

- Age at least 18 years.
- Colorectal cancer with unresectable metastatic unilobar or bilobar liver disease and disease progression in the liver with either (1) oxaliplatin-based or (2) irinotecan-based first-line chemotherapy; patient must be eligible to receive second-line standard-of-care chemotherapy with the alternate regimen. The determination of unresectable was based on local consideration, provided that the treatment decision was made by a multidisciplinary team that included a surgeon.
- Primary tumor either resected or clinically stable.
- Baseline images with measurable target tumors in the liver according to Response Evaluation Criteria in Solid Tumors version 1.1 using standard imaging techniques taken within 28 days before randomization. Images must be taken at completion of first-line chemotherapy or after.
- Tumor replacement less than 50% of total liver volume.
- Eastern Cooperative Oncology Group Performance Status score of 0 to 1 from screening to first treatment on study.
- Laboratory parameters: serum creatinine up to 2.0 mg/dL, serum bilirubin up to 1.2× upper limit of normal, albumin at least 3.0 g/dL, and neutrophil count more than 1200/mm^3^ (1.2 × 10^9^/L).

**Exclusion criteria**

- Prior external beam radiation treatment to the liver and prior intra-arterial liver-directed therapy (including transarterial chemoembolization or TheraSphere yttrium-90 microspheres therapy).
- Planned nonstudy liver-directed therapy or radiation therapy. Planned treatment with biological agents within 28 days before receiving TheraSphere.
- Confirmed extrahepatic metastases. Limited, indeterminate extrahepatic lesions in the lung and/or lymph nodes are permitted (up to 5 lesions in the lung, with each individual lesion smaller than 1 cm; any number of lymph nodes with each individual node smaller than 1.5 cm).
- History of hepatic encephalopathy; history of severe peripheral allergy or intolerance to contrast agents, narcotics, sedatives, or atropine that cannot be managed medically.
- Contraindications to angiography and selective visceral catheterization, such as bleeding diathesis or coagulopathy that is not correctable by usual therapy with hemostatic agents; contraindications to the planned second-line chemotherapy regimen.
- Pulmonary insufficiency or clinically evident chronic obstructive pulmonary disease.
- Cirrhosis or portal hypertension.
- Receipt of intervention for the Ampulla of Vater or compromise thereof.
- Clinically evident ascites aside from trace ascites on imaging.
- Unresolved toxicities related to cancer therapy that the investigator determines will continue and compromise patient safety.
- Significant life-threatening extrahepatic disease, for example, unresolved diarrhea or serious unresolved infections, such as human immunodeficiency virus, acute hepatitis B virus, or hepatitis C virus.

### Randomization and Stratification

Eligible patients are randomized on day 0 in a 1:1 ratio to either the control group or the TARE group. If the study is not stopped early for efficacy, approximately 210 patients will be randomized to each group. To randomize eligible patients, the study site contacts the central randomization office where randomization will be determined using assignment by a computer-generated randomization scheme. Upon randomization, each patient is assigned an identity code. To ensure that treatment groups are balanced, patients are stratified at randomization based on the extent of liver involvement (unilobar vs bilobar disease), type of first-line chemotherapy (oxaliplatin-based vs irinotecan-based), and KRAS status (wild type vs mutant). Additional factors permitting covariate analysis are captured at randomization but are not stratification criteria. These factors have been defined based on the planned covariate analyses. Patients randomized to either the control or the TARE group who are unable to receive their planned study treatment continue to be followed under their assigned study group for the purpose of the intent-to-treat analysis.

### Chemotherapy

**Figure 1:**
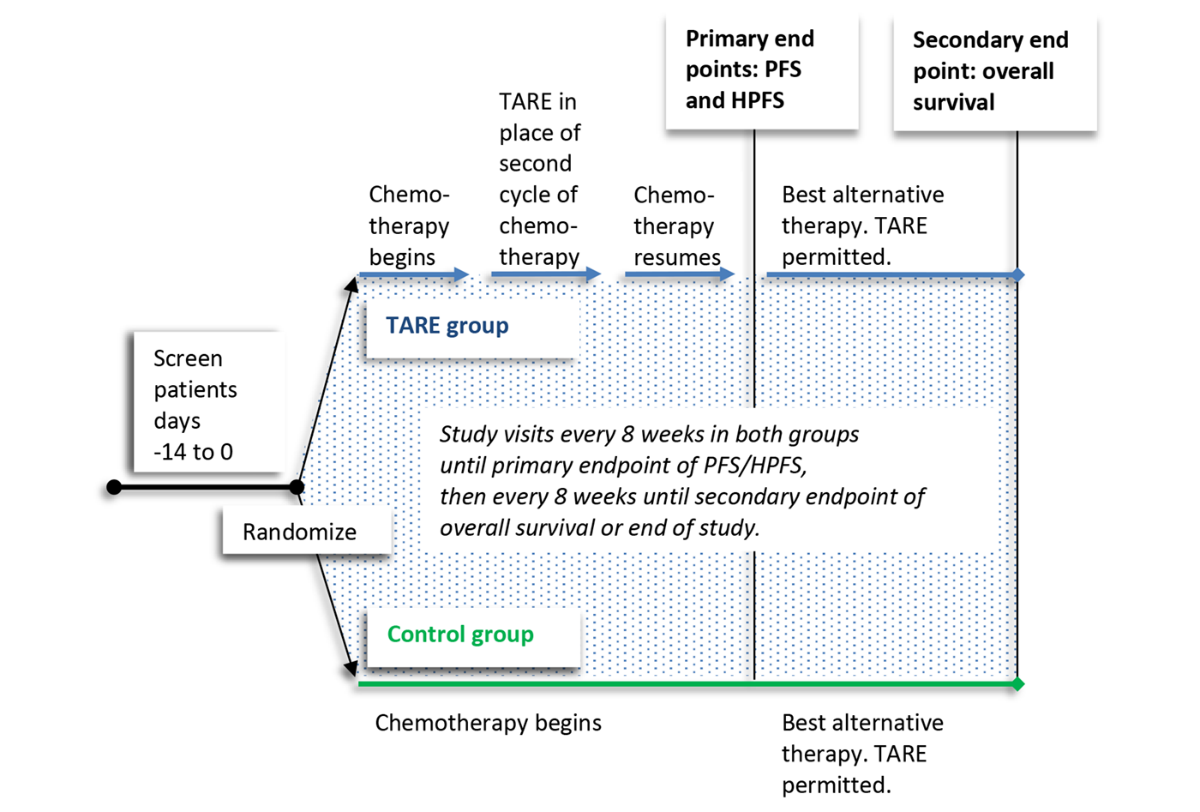
Clinical trial schema for the TheraSphere microspheres EPOCH study. Eligible patients have a washout period of at least 14 days from previous chemotherapy and biologic agents. Second-line chemotherapy is started within 21 days of randomization. Control group: biologic agents are permitted starting at the first cycle of second-line chemotherapy. TARE group: biological agents are discontinued at least 28 days before TARE and are not permitted until the first cycle of second-line chemotherapy that occurs after TARE. HPFS: hepatic progression-free survival; PFS: progression-free survival; TARE: transarterial radioembolization with TheraSpheres™ microspheres.

The treatment schema is shown in Figure 1.

For both groups, second-line chemotherapy started within 21 days of randomization. If during the first-line chemotherapy, patients had received an oxaliplatin-based regimen, then during the EPOCH study, they receive a standard-of-care irinotecan-based chemotherapy. Those who received an irinotecan-based regimen as first-line therapy receive a standard-of-care oxaliplatin-based regimen in this study. Generally, second-line chemotherapy is given every 2 weeks for 6 to 12 cycles; chemotherapy is continued at the investigator’s discretion.

Control group patients can receive biological agents starting with the first cycle of second-line chemotherapy. For the TARE group, one cycle of chemotherapy is administered before the TheraSphere treatment, and biological agents may only be added to the first cycle of chemotherapy regimen that occurs after the TheraSphere administration.

### Transarterial Radioembolization

In the TARE group, TheraSphere microspheres are administered in place of the second cycle of chemotherapy. The treatment approach for TheraSphere can be lobar or selective. Patients with unilobar disease receive TARE to the diseased lobe. Patients with bilobar disease receive treatment to both lobes via successive lobar infusions during the same treatment session. Biological agents must be discontinued for at least 28 days before TARE. Chemotherapy resumes 2 weeks after TARE, at which time treatment with biologic agents may be started.

Eligibility for TARE with TheraSphere microspheres is determined by evaluations that include a pretreatment angiography with technetium-99m macroaggregated albumin (^99m^Tc-MAA), followed by a^99m^Tc-MAA single-photon emission computed tomography (SPECT) or SPECT-CT scan to assess the potential for shunting microspheres to the lungs as well as the potential for the deposition of microspheres to the gastrointestinal tract. Repeat^99m^Tc-MAA may be needed for subsequent treatments to estimate cumulative lung shunt or to reassess gastrointestinal shunting. Patients are ineligible for TARE if radiation exposure to the lungs exceeds 30 Gy (or 50 Gy cumulative across all planned infusions, estimated during the dose calculation) or embolization cannot be performed to effectively block gastrointestinal blood flow from the hepatic arterial system. Randomized patients deemed ineligible for TARE continue with the planned second-line chemotherapy.

Radioembolization can be performed via a segmental or lobar approach, depending on operator preference and angiographic anatomy. The targeted administered dose is 120±10% Gy to the target volume, based on a single-compartment Medical Internal Radiation Dose model [23].

TheraSphere should be administered by appropriately trained or designated personnel from the departments of radiology, nuclear medicine, and/or interventional radiology. Before the activation of trial sites with the appropriate approvals to administer TheraSphere, the health care professionals directly involved in the planning and administration of TheraSphere are required to attend a center of excellence training in which expert speakers lecture on topics related to patient selection and TheraSphere dosimetry and administration. Investigational teams must then accomplish at least three (or five) patient treatment cases with TheraSphere. The minimum number of cases is determined by a trained team of study sponsor proctors who review the treatment planning and perform on-site review of treatment administration. Upon completion of the training program, investigators are trained on the study protocol.

### Treatment for Disease Progression

After disease progression is observed and confirmed, patients in either group may receive the best alternative therapy or care that the investigator considers appropriate. Patients in the TARE group who have hepatic progression with hepatic lesions that are still amenable to TheraSphere are eligible for repeat treatment with TheraSphere. In these patients, TheraSphere may be administered at the investigator’s discretion on separate treatment days. Although this is not a cross-over study, patients in the control group who have hepatic progression with hepatic lesions that are amenable to liver-directed therapy can receive TheraSphere or any other liver-directed therapies according to the investigator’s decision. As with the initial administration of TheraSphere, biological agents must be discontinued for at least 28 days before any additional TheraSphere administration.

### Follow-Up Evaluations

The schedule of postrandomization evaluations is shown in Table 1.

Efficacy assessment scans are taken according to standard-of-care clinical management guidelines every 8 weeks (±1 week) after randomization until either death, withdrawal from study follow-up, or end of the study. The imaging modality used at baseline must be used throughout the study. Image assessment must follow study imaging guidelines. Tumor response is evaluated locally according to RECIST version 1.1. In case of progression, a confirmatory scan is requested. If the first progression occurs outside the liver, efforts must be made to continue the scheduled follow-up until hepatic progression occurs; no confirmatory scan is requested. Imaging performed for disease assessment must be submitted to the sponsor or designate for centralized review. Adverse events, serious adverse events, and unanticipated adverse device effects, as defined by the study protocol, will be collected throughout the study.

Independent review of the CT and MRI images is performed by a central imaging review organization. Central image review interpretation (independent reads by 2 radiologists with adjudication, if required, by a third radiologist) is performed in a blinded fashion on the full set of patient images and captured on an electronic case report form. The results of the primary study end points will be based on the central image review findings.

### Efficacy End Points and Definitions

The primary efficacy end points of the EPOCH study are progression-free survival and hepatic progression-free survival. The secondary efficacy end points are overall survival, time to symptomatic progression, objective response rate, disease control rate, quality of life assessment by FACT-C questionnaire, and adverse events. Definitions of these outcomes are as follows:

1. Progression-free survival is the time from the randomization date to the date of radiological progression or death from any cause, whichever occurs first. Radiological progression is determined by blinded central image review according to RECIST version 1.1.
2. Hepatic progression-free survival is the time from randomization to the date of radiological progression in the liver or death from any cause, whichever occurs first. Radiological progression is determined by blinded central image review according to RECIST version 1.1.
3. Overall survival is the time from the randomization date to death from any cause.
4. Time to symptomatic progression is the time from randomization to ECOG Performance Status score greater than 2 points. Such deterioration in performance score is to be confirmed at one subsequent evaluation at least 8 weeks later.
5. Objective response rate is the proportion of patients achieving a best tumor response of either complete response or partial response during the study, as assessed by blinded central image review according to RECIST version 1.1.
6. Disease control rate is the proportion of patients achieving a best tumor response of either complete response, partial response, or stable disease during the study, as assessed by blinded central image review according to RECIST version 1.1.
7. Quality-of-life assessment is based on the patient-reported FACT-C questionnaire. Deterioration in quality of life is a decline of at least seven points in the total FACT-C score or death, whichever occurs first. The time to deterioration in quality of life is calculated as the time from randomization to deterioration in quality of life.

TARE can cause tumor inflammation (edema) early after treatment; therefore, any tumor assessments performed within six weeks of randomization will not be included in the analysis of imaging-related efficacy end points to rule out the risk of false progression.

**Table 1:** Schedule of events after randomization on day 0.

Interventions and assessments		Chemotherapy	First TARE^a^ workup and administration in TARE group	Study visits to progression	Additional TARE workup and administration^b^	Study visits until death or end of study
Description		Every 2 weeks	Replaces second cycle of chemotherapy	Every 8 weeks from day 0 (±1 week)	After hepatic progression, TARE replaces a cycle of chemotherapy	Every 8 weeks (±1 week)
**Interventions**						
	Hepatic angiogram,^99^^m^Tc-MAA^c^ scan,^d^ calculate liver volume and mass,^d^ calculate TheraSphere dose,^d^ order and administer TheraSphere	—^e^	✓	—	✓	—
	Administer second-line chemotherapy	✓	—	—	—	—
**Assessments**						
	ECOG^f^ Performance Status	✓^g^	✓	✓	✓	✓^g^
	Hematology^h^, chemistry panel, liver function tests	✓	—	✓	✓	—
	Coagulation tests^i^	✓^j^	—	—	✓	—
	Serum pregnancy^k^	—	✓	—	✓	—
	Tumor markers for colorectal cancer (serum carcinoembryonic antigen)	—	—	✓	—	—
	Record and administer any chemotherapy following second-line chemotherapy^l^	—	—	—	—	✓
	Quality-of-life questionnaire	—	—	✓	—	✓^g^
	Spiral CT^m^ or MRI^n^ of the abdomen, pelvis, or chest^o^	—	—	✓	✓	—
	Assess and report adverse events	✓	✓	✓	✓	✓
	Review and record concurrent medication	✓	✓	✓	✓	✓
	Final end point, efficacy and safety documentation, and exit patient	—	—	—	—	✓

^a^TARE: transarterial radioembolization.

^b^In lesions amenable to further TARE.

^c^^99m^Tc-MAA: technetium-99m macroaggregated albumin.

^d^Before TARE administration.

^e^Assessment or intervention was not conducted at that time.

^f^ECOG: Eastern Cooperative Oncology Group.

^g^Can be done remotely if patient is not coming in for clinic visit.

^h^Hematology tests: white blood cells with differential, hemoglobin, hematocrit, and platelets.

^i^Coagulation tests: prothrombin time, partial thromboplastin time, and international normalized ratio.

^j^Only required at chemotherapy visits as clinically indicated, that is, if patient is being followed for coagulopathy.

^k^Required for female patients of childbearing potential.

^l^All randomized patients must receive chemotherapy within 21 days of randomization.

^m^CT: computed tomography.

^n^MRI: magnetic resonance imaging.

^o^All attempts should be made to obtain imaging every 8 weeks until hepatic progression, plus confirmatory scan.

The following additional efficacy variables will be assessed:

- Progression-free survival and hepatic progression-free survival by investigator assessment, with progression determined by the investigator according to RECIST version 1.1.
- Objective response rate, determined by the investigator, defined as the proportion of randomized patients achieving a best overall response of complete response or partial response, as defined by RECIST version 1.1.
- Duration of objective response will be determined for patients who had a best response of complete response or partial response. Duration of objective response is defined as the time from the first date of complete response or partial response to date of progression or death from any cause, whichever occurs first. Duration of response will be assessed separately for response determined by blinded central image review and by investigator assessment.
- Disease control rate, as determined by the investigator, defined as the proportion of randomized patients achieving a best overall response of complete response, partial response, or stable disease as defined by RECIST version 1.1.
- Duration of disease control will be determined for patients who had a best response of complete response, partial response, or stable disease. Duration of disease control is defined as the time from the first date of complete response, partial response, or stable disease until the date of progression or death from any cause, whichever occurred first. Duration of disease control will be assessed separately for response determined by blinded central image review and by investigator assessment.
- Depth of response, defined as the percentage change from baseline to the nadir in the sum of the longest diameters of target lesions.
- Posttreatment tumor shrinkage, defined as the proportion of patients achieving a decrease of at least 20% in the sum of the longest diameters of target lesions.
- Change from baseline in tumor marker for colorectal cancer (carcinoembryonic antigen).

### Planned Statistical Analysis

#### Sample Size Estimate

The study is a phase 3 adaptive trial, comprising a group sequential design with 2 interim analyses. The study could be stopped early for efficacy at an interim analysis based on superiority in progression-free survival but not hepatic progression-free survival.

The study is designed to detect a 2.5-month increase in median progression-free survival, from 6 months in the control group to 8.5 months in the TARE group (hazard ratio [HR] 0.71), and a 3.5-month increase in median hepatic progression-free survival time, from 6.5 months in the control group to 10 months in the TARE group (HR 0.65). On the basis of simulations, the power to detect the target difference in either progression-free survival or hepatic progression-free survival is >90%, and the power to detect the target difference in each end point alone is >80%, using log-rank tests.

The analysis of progression-free survival will be based on a group sequential design with 2 interim analyses at 50% and 70% of the required total number of 344 progression-free survival events with a stopping boundary defined by the rho family error spending function with ρ=1.5 [24]. It is estimated that a maximum of 420 patients will need to be recruited over 36 months, with a 1-year additional follow-up period, allowing for 10% of patients lost to follow-up and for whom a date of progression or death is not recorded. Although the forecasted accrual period has been increased to 60 months, this does not increase the number of patients required or affect the statistical power of the study because both the power and the timing of the interim and final analyses are based on the number of progression-free survival events rather than the number of patients. The Hochberg procedure will be used to control type 1 error for the 2 primary end points at the final analysis [25].

A simulation study, assuming that progression-free survival and hepatic progression-free survival have a correlation between 0.3 and 0.8, showed that the power to detect the target difference in either median progression-free survival (ie, HR 0.71) or median hepatic progression-free survival (ie, HR 0.65) is >90%, and the power to detect the target difference in progression-free survival or hepatic progression-free survival alone is >80%. The simulation study also demonstrated control of type 1 error at the nominal one-sided level of 0.025.

#### Populations

The intent-to-treat population will comprise all randomized patients. The per-protocol population will be analyzed according to the treatment actually received, excluding patients with major protocol deviations that may affect the efficacy evaluation. The safety analysis population will comprise all randomized patients who received study treatments at least once.

#### Primary Efficacy End Points

##### Analysis of Primary End Points

Progression-free survival and hepatic progression-free survival will be compared between the control and TARE groups using log-rank tests. The HR and 2-sided 95% CI will be computed. Kaplan-Meier curves will also be produced.

##### Interim Analyses of Primary End Point of Progression-Free Survival

The first interim analysis is planned at 172 progression-free survival events. Progression-free survival will be compared between treatment groups using a log-rank test converted to a z-score and compared with the nominal critical value of 2.372 based on the rho family error spending function corresponding to a one-sided *P* ≤.0088, allowing the study to be stopped early for efficacy, in which case hepatic progression-free survival will be tested at the same boundary as progression-free survival using a log-rank test converted to a z-score.

A second interim analysis is planned at 241 progression-free survival events, where progression-free survival will be compared between treatment groups using a log-rank test converted to a z-score and compared with the nominal critical value of 2.330 based on the rho family error spending function corresponding to a one-sided *P* ≤.0099, allowing the study to be stopped early for efficacy. If the study is stopped early for progression-free survival at the second interim analysis, hepatic progression-free survival will be tested using the boundary derived based on an incremental alpha of .0057. This boundary will account for the correlation between the z-score for progression-free survival at the first interim analysis and the z-score for hepatic progression-free survival at the second interim analysis, which is determined by the observed number of hepatic progression-free survival events at the first interim analysis and the cumulative number of hepatic progression-free survival events observed at the second interim analysis.

##### Final Analysis of Primary End Points of Progression-Free Survival and Hepatic Progression-Free Survival

The final analysis is planned at 344 progression-free survival events. The Hochberg procedure will be used to control type 1 error for the 2 primary end points [25]. Whichever of progression-free survival or hepatic progression-free survival that has the larger *P* value will be compared between treatment groups using a log-rank test converted to a z-score and compared with the nominal critical value of 2.312 with a corresponding one-sided *P* ≤.0104 required to declare a statistically significant improvement in hazard rate for this end point. To ensure that type 1 error is controlled for both primary end points, this boundary is based on the incremental alpha of .0104 instead of the *P* value scale boundary of .0168, using the rho family error spending function with ρ=1.5.

According to the Hochberg procedure, if the primary end point with the larger *P* value is statistically significant, then the other primary end point is also statistically significant. However, if the primary end point with the larger *P* value is not statistically significant, then the other primary end point will be compared between treatment arms using a log-rank test converted to a z-score and compared with the nominal critical value of 2.562 based on the rho family error spending function, with a corresponding one-sided *P* ≤.0104/2=.0052 required to declare a statistically significant improvement in hazard rate for this end point.

#### Analysis of Secondary Efficacy End Points

Comparison between treatment groups for all secondary end points will be conducted at the final analysis at one-sided alpha of .025.

Time-to-event end points (ie, overall survival, time to symptomatic progression, and time to deterioration in quality of life) will be compared between treatment groups using a log-rank test. Disease control rates and objective response rate will be compared between treatment groups using the continuity-adjusted Newcombe-Wilson test. The FACT-C score will be compared between treatment arms using a mixed linear model with baseline score and the relative time from baseline as covariates.

#### Poolability and Other Analyses

Univariable Cox regression analyses of the primary efficacy end points and all other time-to-event end points (ie, overall survival, time to symptomatic progression, and time to deterioration in quality of life) will be conducted with the following baseline factors, one at a time, together with randomized group: age group, race, ethnicity, gender, unilobar versus bilobar disease, oxaliplatin or irinotecan first-line chemotherapy, KRAS status, ECOG performance status, region, duration of time from date of diagnosis of mCRC to randomization, duration of time from the start of first-line chemotherapy to date of progression on first-line therapy, duration of time from date of progression on first-line therapy to date of start of second-line chemotherapy, synchronous versus metachronous metastases, location of primary tumor at the time of first diagnosis of primary colorectal cancer (right-sided vs left-sided), tumor burden, and presence of lung or lymph node lesions. Receipt of oxaliplatin- or irinotecan-based second-line chemotherapy and the receipt of biological agents during the study will also be assessed one at a time. This will allow an assessment of each of these factors on the study outcomes.

To assess the poolability of data across study sites, multivariable Cox regression analyses of the time-to-event end points will be conducted, including factors of randomized group, study site, randomized group by study site interaction, and the factors from the univariable analyses that have a one-sided *P*<.075. Similarly, to assess the poolability of data across regions, Cox regression analysis will be conducted with study site replaced by region.

Logistic regression analyses of the binary end points (ie, objective response rate and disease control rate) will be conducted in the same way as the Cox regression analyses described above.

## Results

After the first 20 patients in the TARE group received TheraSphere microspheres followed by at least two cycles of chemotherapy, a feasibility safety assessment was conducted. The IDMC reviewed the safety results of both groups in an unblinded fashion. A consideration for adjusting the dose of cytotoxic agents, other safety recommendations, or stopping further enrollment could have been made if there was either an unanticipated patient death definitely or probably related to the sequential administration of TARE with oxaliplatin-based or irinotecan-based chemotherapy or there was a pattern of serious toxicity clearly related to the sequential administration of TARE with oxaliplatin-based or irinotecan-based chemotherapy. However, the IDMC did not recommend any changes to the study.

Enrollment for the study completed in October 2018. Results of the first interim analysis were reviewed by the IDMC, and their recommendation was to continue the study without any changes.

## Discussion

### Overview

First-line chemotherapy regimens for unresectable mCRC are well established [10]. Second-line therapy using the combination of chemotherapy and targeted therapies has demonstrated efﬁcacy with improved overall survival, progression-free survival, and overall response rate in comparison with chemotherapy alone [26,27]. However, the choice of the optimal treatment strategy remains challenging and is mostly driven by the type of and response to the first-line treatment administered, the type of retrovirus-associated DNA sequence mutation (KRAS, NRAS, and HRAS), microsatellite stability, tumor burden, patient performance status, and comorbidities.

The EPOCH study was designed to account for known prognostic and predictive factors [10]. Patients in the EPOCH study are stratified at randomization according to key factors that could influence the primary or secondary study end points: (1) tumor load (unilobar vs bilobar), (2) KRAS status (wild type vs mutant), and (3) prior first-line chemotherapy (oxaliplatin-based or irinotecan-based) to ensure balance between groups.

High tumor load is a known factor of chemotherapy failure [28]. KRAS mutation is a predictive factor of nonresponse to epidermal growth factor receptor inhibitor treatment [29,30]. Whether KRAS mutation is a prognostic factor is widely debated. KRAS mutant patients may have different sensitivity to treatment compared with KRAS wild-type patients [31-35], and KRAS mutational status will drive the postprogression treatment received. Second-line treatment with irinotecan-based regimens has been associated with shorter median progression-free survival [36]. Tumor sidedness is currently undergoing intense study, given the recognition that tumor location is prognostic and predictive [37-39]. Tumor sidedness is not a stratification factor in the EPOCH study because when the study was designed, the importance of this prognostic factor was less understood; however, a preplanned analysis to assess the impact of this factor will be performed. In addition, the impact of important covariates, such as asynchronous or metachronous metastases, location of primary tumors (right vs left), presence of lung or lymph node lesions at baseline, and biological agents received, will be assessed. This study was designed before well-established data on the prognostic significance of BRAF mutations were available, and thus, BRAF mutation status was not collected on the electronic case report forms. However, because BRAF mutant tumors are often right sided, the preplanned analysis to assess the impact of right-sided vs left-sided primary tumor location should provide an indirect assessment of BRAF mutation status.

TARE treatment for patients with liver or liver-dominant disease has been outlined in prospective and retrospective studies that have demonstrated that this option was manageable in this patient population and compared favorably with standard-of-care treatment [14-16,40,41]. The relevancy of TARE in the first-line setting was extensively explored in the pooled analyses of the randomized studies FOXFIRE, SIRFLOX, and FOXFIRE-Global [42,43]. This analysis demonstrated that TARE (with ^90^Y resin microspheres) in association with an oxaliplatin- and fluorouracil-based chemotherapy regimen failed to improve overall survival and progression-free survival compared with chemotherapy alone. However, the combination of TARE with first-line oxaliplatin-based chemotherapy significantly improved response rate and liver-specific progression-free survival in comparison with chemotherapy alone. In spite of an increased toxicity in the TARE group, the quality of life was not significantly different between the 2 treatment groups [42-44]*.* Factors that may have contributed to the failure of the previous ^90^Y resin microspheres trials are a long delay between randomization to starting TARE treatment, the percentage of patients with extrahepatic metastatic disease at baseline (40% of patients enrolled), the lower percentage of patients in the TARE group who received postprogression therapies, and inferior chemotherapy dose intensity in the TARE group.

In the EPOCH study, the patient population and treatment schedule were selected to avoid these factors that may have contributed to the failure of the previous first-line trials. Patients with rapid and diffuse progression of disease and those with extrahepatic metastasis are not eligible to participate, thus limiting the risk of the trial not reaching a primary progression-free survival end point because of extrahepatic progression. In addition, to avoid a discrepancy between the 2 treatment groups regarding chemotherapy intensity and the delay to start the attributed treatment, the 2 groups must start the first study treatment, that is, chemotherapy, within 21 days after randomization. Both groups also receive an optimal dose of chemotherapy based on oncologist determination. No dose reduction was preplanned in the TARE group. The safety of the treatment schedule was evaluated after the treatment of 20 patients, and the schedule was considered safe by the IDMC. Limitations of the study include the unblinded design, which is required for such a complex treatment intervention when it would be unethical to consider a *sham* procedure for the control group.

### Conclusion

It is important to establish an effective and tolerable treatment to improve patient outcome for unresectable liver metastases from colorectal cancer. One challenge in the treatment of mCRC patients with liver-dominant disease is providing an efficient treatment of the cancer without impairing liver function and allowing systemic treatment to continue with minimal interruption. TARE has a limited toxicity profile when used appropriately and, consequently, a low impact on the dose intensity and duration of systemic treatment. Enrollment for the EPOCH study completed in October 2018. Data from this trial will enhance the knowledge base regarding optimal treatment options for patients with unresectable mCRC.

## Appendix

Multimedia Appendix 1 IRB approval letter for the principal investigator of the multicenter study.

## Abbreviations

^90^Y: yttrium-90
^99m^Tc-MAA: technetium-99m macroaggregated albumin
CT: computerized tomography
ECOG: Eastern Cooperative Oncology Group
EPOCH: A Phase 3 Clinical Trial Evaluating TheraSphere in Patients with Metastatic Colorectal Carcinoma of the Liver who have Failed First Line Chemotherapy
FACT-C: Functional Assessment of Cancer Therapy-Colorectal Cancer
HR: hazard ratio
IDMC: independent data monitoring committee
KRAS: Kirsten retrovirus–associated DNA sequence
mCRC: metastatic colorectal cancer
MRI: magnetic resonance imaging
RECIST: Response Evaluation Criteria in Solid Tumors
SPECT: single-photon emission computed tomography
TACE: transarterial chemoembolization
TARE: transarterial radioembolization
