# Supplementary material for: TheraSphere Yttrium-90 Glass Microspheres Combined With Chemotherapy Versus Chemotherapy Alone in Second-Line Treatment of Patients With Metastatic Colorectal Carcinoma of the Liver: Protocol for the EPOCH Phase 3 Randomized Clinical Trial
Source: JMIR Res Protoc. 2019 Jan 17;8(1):e11545. doi: 10.2196/11545 (PMC6354199; doi:10.2196/11545)
Supplement: Multimedia Appendix 1 [file resprot_v8i1e11545_app1.pdf]

Institutional Review Board Office
Northwestern University

Biomedical IRB
750 North Lake Shore Drive
Suite 700
Chicago, Illinois 60611
312-503-9338

Social and Behavioral Sciences IRB
2205 Tech Drive
Hogan Bldg. Suite G-100
Evanston, Illinois 60208
847-467-1723

NORTHWESTERN
UNIVERSITY

4/5/2012

Dr. Mary Mulcahy

Hematology/Oncology Division
676 North Saint Clair Suite 850
Chicago,IL 60611
m-mulcahy@northwestern.edu

IRB Project Number: STU00059821
Project Title: DRUG TS-102: A Phase III Clinical Trial Evaluating TheraSphere® in Patients with Metastatic Colorectal Carcinoma of the Liver who have Failed First Line Chemotherapy
Project Sites:

Northwestern Medical Faculty Foundation (NMFF)
* Robert H. Lurie Comprehensive Cancer Center
Northwestern University (NU)
Affiliate sites of Robert H. Lurie Comprehensive Cancer Center (You must also select Robert H. Lurie Comprehensive Cancer Center)
Northwestern Memorial Hospital (NMH)

Sponsor Information (Grant #, if applicable):

View	Nordion Inc

Submission Considered: New Submission **Submission Number:** STU00059821
Study Review Type: Full IRB Review
Meeting Date: 03/02/2012 **Panel:** Panel A
Status: APPROVED **Approval Period:** (4/5/2012 - 3/1/2013)

Dear Dr. Mulcahy,

The IRB considered and approved your submission referenced above through 3/1/2013 . As Principal Investigator (P.I.), you have ultimate responsibility for the conduct of this study, the ethical performance of the project, and the protection of the rights and welfare of human subjects. You are required to comply with all NU policies and procedures, as well as with all applicable Federal, State and local laws regarding the protection of human subjects in research including, but not limited to the following:

- Not changing the approved protocol or consent form without prior IRB approval (except in an emergency, if necessary, to safeguard the well-being of human subjects).

- Obtaining proper informed consent from human subjects or their legally responsible representative, using only the currently approved, stamped consent form.
- Promptly reporting unanticipated problems involving risks to subjects or others, or promptly reportable non-compliance in accordance with IRB guidelines.
- Submit a continuing review application 45 days prior to the expiration of IRB approval. If IRB re-approval is not obtained by the end of the approval period indicated above, all research related activities must stop and no new subjects may be enrolled.

IRB approval includes the following:

Written Consent Form/Consent Form and Authorization for Research:

Name
DRUG TS-102 ICF dated 03.29.2012.doc

Protocol Document:

Name
DRUG TS-102 Protocol dated 11.18.11 v2.0.pdf

Recruitment Materials:

Name
DRUG TS 102 draft abstract.docx

Survey/Questionnaires:

Name
FACT-C ENG Final Ver4_16Nov07.pdf

For more information regarding IRB Office submissions and guidelines, please consult http://www.northwestern.edu/research/OPRS/irb.
This Institution has an approved Federalwide Assurance with the Department of Health and Human Services: FWA00001549.

Institutional Review Board Office
Northwestern University
Biomedical IRB
750 North Lake Shore Drive
Suite 700
Chicago, Illinois 60611
312-503-9338

Social and Behavioral Sciences IRB
600 Foster Street
Chambers Hall, Second Floor
Evanston, Illinois 60208
847-467-1723

5/10/2012

Dr. Mary Mulcahy

Hematology/Oncology Division
676 North Saint Clair Suite 850
Chicago,IL 60611
m-mulcahy@northwestern.edu

IRB Project Number: STU00059821
Project Title: DRUG TS-102: A Phase III Clinical Trial Evaluating TheraSphere® in Patients with Metastatic Colorectal Carcinoma of the Liver who have Failed First Line Chemotherapy
Project Sites:

Northwestern Medical Faculty Foundation (NMFF)
* Robert H. Lurie Comprehensive Cancer Center
Northwestern University (NU)
Affiliate sites of Robert H. Lurie Comprehensive Cancer Center (You must also select Robert H. Lurie Comprehensive Cancer Center)
Northwestern Memorial Hospital (NMH)

Sponsor Information (Grant #, if applicable):

View Nordion Inc

Submission Considered: New Submission **Submission Number:** STU00059821
Study Review Type: Full IRB Review
Meeting Date: 03/02/2012 Panel: A
Status: APPROVED **Approval Period:** (4/5/2012 - 3/1/2013)

Dear Dr. Mulcahy,

The IRB considered and approved your submission referenced above through 3/1/2013 . As Principal Investigator (P.I.), you have ultimate responsibility for the conduct of this study, the ethical performance of the project, and the protection of the rights and welfare of human subjects. You are required to comply with all NU policies and procedures, as well as with all applicable Federal, State and local laws regarding the protection of human subjects in research including, but not limited to the following:

- Not changing the approved protocol or consent form without prior IRB approval (except in an

emergency, if necessary, to safeguard the well-being of human subjects).

- Obtaining proper informed consent from human subjects or their legally responsible representative, using only the currently approved, stamped consent form.
- Promptly reporting unanticipated problems involving risks to subjects or others, or promptly reportable non-compliance in accordance with IRB guidelines.
- Submit a continuing review application 45 days prior to the expiration of IRB approval. If IRB re-approval is not obtained by the end of the approval period indicated above, all research related activities must stop and no new subjects may be enrolled.

IRB approval includes the following:

Written Consent Form/Consent Form and Authorization for Research:
Name
DRUG TS-102 ICF dated 03.29.2012.doc

Protocol Document:
Name
DRUG TS-102 Protocol version 1.0 dated 3-7-2011

Recruitment Materials:
Name
DRUG TS 102 draft abstract.docx

Survey/Questionnaires:
Name
FACT-C ENG Final Ver4_16Nov07.pdf

For more information regarding IRB Office submissions and guidelines, please consult http://www.northwestern.edu/research/OPRS/irb.
This Institution has an approved Federalwide Assurance with the Department of Health and Human Services: FWA00001549.

This letter supercedes the approval letter dated 4/5/2012. This letter has been updated to reflect the correct protocol date, version 1.0 dated 3/7/2011
